# Characterization of cardiac involvement in patients with *LMNA* splice-site mutation–related dilated cardiomyopathy and sudden cardiac death

**DOI:** 10.3389/fgene.2023.1291411

**Published:** 2024-01-08

**Authors:** Xuebin Ling, Yanjun Hou, Xingyu Jia, Youling Lan, Xiaoping Wu, Julan Wu, Wei Jie, Hui Liu, Shan Huang, Zhenling Wan, Tianfa Li, Junli Guo, Tiebiao Liang

**Affiliations:** ^1^ Department of Cardiovascular Medicine and Hainan Provincial Key Laboratory for Tropical Cardiovascular Diseases Research and Hainan Engineering Research Center for Biological Sample Resources of Major Diseases, The First Affiliated Hospital of Hainan Medical University, Haikou, China; ^2^ Department of Cardiovascular Surgery, The Second Affiliated Hospital of Hainan Medical University, Haikou, China; ^3^ Department of Pathology, Hainan Women and Children Medical Center, Hainan Medical University, Haikou, China; ^4^ Department of Cardiovascular Medicine, People’s Hospital of Wanning, Wanning, China

**Keywords:** LMNA, lamin A/C, desmin, connexin 43, dilated cardiomyopathy (DCM)

## Abstract

**Introduction:** *LMNA* splicing mutations occur in 9.1% of cases with cardiac involvement cases, but the phenotype and severity of disease they cause have not yet been systematically studied. The aim of this study was to understand the clinical and pathogenic characteristics of the *LMNA* splice-site mutation phenotype in patients with *LMNA*-related dilated cardiomyopathy (DCM) and sudden cardiac death (SCD).

**Methods and Results:** First, we reported a novel family with *LMNA*-related DCM and SCD, and the clinical characteristics of all current patients with *LMNA* splicing mutations were further summarized through the ClinVar database. Seventeen families with a total of 134 individuals, containing a total of 15 *LMNA* splicing mutation sites, were enrolled. A total of 42 subjects (31.3%) had SCD. Compared without with the non-DCM group (*n* = 56), the patients within the DCM group (*n* = 78) presented a lower incidence of atrioventricular block (AVB) (*p* = 0.015) and a higher incidence rates of non-sustained ventricular tachycardia (*p* = 0.004),) and implantable cardioverter defibrillator (ICD) implantation (*p* = 0.005). Kaplan‒Meier survival analysis showed that the patients with pacemaker (PM) implantation had a significantly reduced the occurrence of SCD compared to patientswith those without PM implantation (log-rank *p* < 0.001), while there was no significant difference in ICD implantation between the two groups (log-rank *p* = 0.73). Second, we identified the family that we reported with a mutation in an LMNA c.513+1 G>A mutation in the reported family, and pathogenic prediction analysis showed that the mutation site was extremely harmful. Next, we conducted gene expression levels and cardiac pathological biopsy studies on the proband of this family. We found that the expression of normal *LMNA* mRNA from the proband was significantly downregulated in peripheral blood mononuclear cells than incompared with healthy individuals. Finally, we comprehensively summarized the pathological characteristics of *LMNA*-related DCM, including hypertrophy, atrophy, fibrosis, white blood cell infiltration, intercalated disc remodeling, and downregulation of desmin and connexin 43 (Cx43) expression.

**Discussion:** Above all, Cardiaccardiac involvement in patients with *LMNA* splice-site mutation presented with a high rate of SCD. Implanting a pacemaker significantly reduced the SCD rate in non-DCM patients with AVB. The pathogenic characterization was not only haveinvolved suppressed the expression of the healthy *LMNA* allele, but was also associated with abnormal expression and distribution of desmin and Cx43.

## Introduction

Lamin A/C, encoded by the *LMNA* gene, is widely expressed in the myocardium and skeletal muscle and plays key roles in cell signal transduction, post-translational modification, maintaining DNA stability in cells, and regulating apoptosis (Meune et al., 2006). *LMNA* produces two kinds of mRNA from exon 10 during transcription, encoding lamin A and lamin C. However, exon missense mutations and intron splice site mutations can cause changes in the mRNA sequence and affect the structure and function of lamin A/C. So far, more than 500 mutation forms and 300 protein variants have been identified (Wang and Dobreva, 2023), which can present a variety of clinical phenotypes, such as dilated cardiomyopathy (DCM) (Lee et al., 2019), Hutchinson–Gilford progeria syndrome (Koblan et al., 2021), and *LMNA*-related muscular dystrophy type 1B (LGMD1B) (Cesar et al., 2023), among others. According to statistics (Captur et al., 2018), 10% of genetically related DCM cases are caused by *LMNA* mutations. *LMNA*-related DCM progresses rapidly, with a sudden cardiac death (SCD) rate as high as 46%, which is related to uncontrollable heart failure and malignant arrhythmia (complete atrioventricular block, ventricular tachycardia, ventricular fibrillation). A 44-point mutation identified in 12 exons of *LMNA* can cause DCM with cardiac conduction system disease (CCD) (Perrot et al., 2009). Splicing mutations in the intron region of *LMNA* mostly cause limb-girdle muscular dystrophy type 1B (LGMD1B) (Cesar et al., 2023; Rogozhina et al., 2013; Chen et al., 2013). Additionally, the combined cardiac involvement is manifested as CCD, which has been reported to include c.513 + 45T>G (Rogozhina et al., 2013) and c.513 + 1G>A (Chen et al., 2013).

The pathogenesis of myocardial lesions caused by *LMNA* mutations is currently unclear. lamin A/C belongs to the type 5 intermediate filament (IF) protein, and desmin is a type 3 intermediate filament protein, both of which form an IF network to jointly maintain cell morphology and function. Evidence has shown that the depletion of desmin causes unfolding of the nuclear envelope, resulting in the loss of nuclear integrity and DNA damage (Heffler et al., 2020). Furthermore, lamin A/C gene mutation in the *LMNA* ^H222P/H222P^ mouse model has been shown to present as desmin aggregation, disorganization of intercalated discs, and mitochondrial defects (Galata et al., 2018). Additionally, intercalated disc dysfunction is the basis for the occurrence of various types of arrhythmia in DCM (Domínguez et al., 2018). It has also been shown that *LMNA* E82K significantly reduces connexin 43 (Cx43) expression and alters its localization in neonatal myocytes (Sun et al., 2010). Cx43 is an important protein expressed in intercalated discs, and it is speculated that *LMNA* mutations can affect cardiac intercalated disc function. However, there is currently a lack of direct evidence of intercalated disc pathological changes in *LMNA*-related DCM.

Here, we report a family with *LMNA* c.513 + 1G>A presenting with DCM combined with malignant ventricular tachycardia without LGMD1B involvement. We comprehensively summarized the clinical characteristics of multiple *LMNA* splicing mutation sites. Furthermore, we explored the pathogenesis of cardiomyopathy from the perspective of abnormal expression of cardiac skeletal proteins, with the aim to provide direct evidence of myocardial remodelling and electrical activity disorders in *LMNA*-related DCM.

## Methods

### Clinical evaluation

The genealogical information of three generations of the study family (from Liaoning province, PR, China) was collected. All subjects underwent an initial clinical evaluation, which included clinical history, encompassing a family history of SCD, cardiogenic death, cardiac arrest, atrioventricular block, dizziness, syncope, cardiac device implantation, muscle strength, and cardiopulmonary examination. Blood routine tests, including plasma potassium, creatine kinase, alanine aminotransferase, and serum creatinine measurements, were performed, and 12-lead electrocardiogram (ECG) and Holter were applied to investigate the presence of sick sinus syndrome, atrioventricular block, bundle branch block, or another arrhythmia. The proband also underwent transthoracic echocardiography. The left atrial and ventricle diameters were obtained in the parasternal long-axis view during end-systole, and the left ventricular ejection fraction was measured by the modified Simpson method.

To further understand the effect of *LMNA* splicing mutations on cardiac phenotype, splicing variants of *LMNA* were identified in cardiomyopathy patients reported to date by the ClinVar database and the human gene mutation database (HGMD). By reviewing the clinical information of probands and family members from previous *LMNA* splicing mutation family reports, we extracted the following information: gender, age of onset, type of arrhythmia, time of pacemaker (PM)/ICD implantation, SCD time, and mutation sites in each family. Finally, the aggregation of all *LMNA*-related cardiac phenotypes across the databases included 23 splicing variants. After excluding cases without available clinical information on family members, we included a total of 15 *LMNA* splicing mutation sites, present in 134 individuals in 17 families (Supplementary Table S1). All variants used for the study are publicly available online at ClinVar (www.ncbi.nlm.nih.gov/clinvar/) and HGMD (https://www.hgmd.cf.ac.uk/ac/).

### Whole-exon sequencing and sanger sequencing

After obtaining the peripheral blood samples of the proband within our reported family, high-throughput sequencing was performed using the HiSeq X system (Illumina, Carlsbad, CA, United States). Genomic DNA was isolated from the blood using a blood genomic DNA extraction kit (Qiagen, Hilden, Germany). PCR amplification was conducted with CAP-PCR Master Mix and CAP Hotstar enzyme, and then purified using Ampure XP beads (AMP, Germany). The following PCR primers were used: 5′-GACCTGACCA TCTGGAGTTGC-3' (forward) and 5′-AGT​AAT​CTC​GAG​CCT​CCT​GGG-3' (reverse). Finally, the results of Sanger sequencing were obtained through ABI PRISM 3730 genetic analysis (Thermo Fisher Scientific, Waltham, MA, United States).

### RNA extraction and reverse transcription–polymerase chain reaction

RNA was extracted from peripheral blood samples of the proband and other six healthy individuals using an RNA Isolation Kit (Roche, United States). Subsequently, cDNA was synthesized using a Transcriptor First Strand cDNA Synthesis Kit (Roche, United States). PCR with cDNA and Sanger sequencing was performed with the primers in exon 1 (1F: 5′- AGC​AAA​GTG​CGT​GAG​GAG​TT -3´; 5′- CCG​AGT​CTG​AAG​AGG​TGG​TC-3′), exon 3 (3R: 5′-GAA​GTT​GCT​TCT​TGG​CCT​CA-3′), and exon 4 (4R: 5′- AAT​CTC​CAC​CAG​TCG​GGT​CT-3′), before sequencing by Tian Yihui Gene Technology (Guangzhou, China) using an ABI3730XL sequencer.

### Real-time quantitative PCR

Real-time quantitative PCR (qPCR) was performed using SYBR Green real-time PCR master mix (Thermos, United States). To determine the transcriptional expression level of *LMNA*, we performed qPCR on the proband and six healthy individuals with specific primers in exons 6–7 (6F: 5′-TGG​ACG​AGT​ACC​AGG​AGC​TT-3´; 7R: 5′-AGT​TTG​CGC​TTT​TTG​GTG​AC-3′); and exons 11–12 (11F: 5′-GCT​CTT​CTG​CCT​CCA​GTG​TC-3´; 12R: 5′-GGG​TTA​TTT​TTC​TTT​GGC​TTC​A-3′). Cycle threshold (Ct) values were recorded for each gene, and target mRNA levels were normalized to the level of glyceraldehyde 3-phosphate dehydrogenase (GAPDH). Relative gene expression was calculated using the 2^−ΔΔCT^ method.

### Mutation and bioinformatics analyses

The impact of candidate sites on splicing effects were predicted using the dbscSNV_ Ada_ Score and dbscSNV_ RF_ Score tools, with scores more than 0.6 indicating the presence of harmful splicing effects (Li and Wang, 2017). Splice AI software is based on deep learning to predict splicing changes caused by single nucleotide mutations, and can accurately predict splicing sites (position and probability of abnormal splicing) from any mRNA precursor sequence, as well as predict abnormal splicing caused by mutations in noncoding RNA regions. The Python package (Inc., Deep Genomics, Canada), download from https://github.com/Illumina/SpliceAI, can predict the sites within 300 bp of splice site to determine whether single nucleotide variation (SNV) affects splicing, and analyze synonymous, missense, and nonsense mutations in the exon region. The SPIDEX annotation database for use in ANNOVAR is downloaded from http://www.openbioinformatics.org/annovar/spidex_download_form.php, and predictive output |z |> = 2 indicates that splicing effects are harmful and destructive.

### Pathological staining: immunohistochemistry and immunofluorescence

A pathological biopsy was performed on the proband’s heart following heart transplantation, while the healthy donor heart that was unsuccessfully used in the first transplant was used as the control group. To observe the general composition and characteristics of the myocardium involvement, 3–5-μm-thick samples were stained by classic histochemical methods, including hematoxylin and eosin (H&E) and Masson’s trichrome (for connective tissue). Human monoclonal antibodies for lamin A/C (Affinit, United States), DES (Abcam, Cambridge United Kingdom), CD45 (Abcam, Cambridge United Kingdom), and Cx43 (Abcam, Cambridge United Kingdom) were used to identify muscle cells by immunohistochemical and polychromatic immunofluorescence staining. As previously described (Li et al., 2019), the Opal 7-Color Manual IHC Kit (catalog no. NEL801001KT, Perkin Elmer, Waltham, MA, United States) was used for the analysis of expression and localization of cytoskeletal proteins in myocardial cells in accordance with the manufacturer’s protocol. The dilution ratio and labeled fluorescence of the antibodies are shown in Supplementary Table S1. After washing with phosphate-buffered saline, DAPI (Beyotime Biotechnology, China) was added and image analysis was performed using a laser confocal microscope (FV3000; Olympus Corporation, Japan). All sections were analyzed by two observers blinded to the study.

### Statistical analysis

For quantitative analysis, Image-Pro Plus 6.0 software (Inc., Media Cybernetics, United States) was applied to the areas showing positive proteins in immunohistochemistry using the integrated optical density (IOD)/area index and the area of collagen fibers using the volume fraction of collagen in Masson’s trichrome–stained sections. We used mean values and Student’s t*-*test to analyse baseline data between two groups. Data on the clinical characteristics were analyzed using Fisher’s exact test (two-sided). Kaplan–Meier survival analysis with a log-rank test was used for comparing the age at diagnosis of DCM or PM/ICD implantation. The data were analyzed using GraphPad Prism 9.0 (GraphPad Software, Inc., San Diego, CA, United States). *p* values <0.05 were considered statistically significant.

## Results

### Clinical case

**FIGURE 1:**
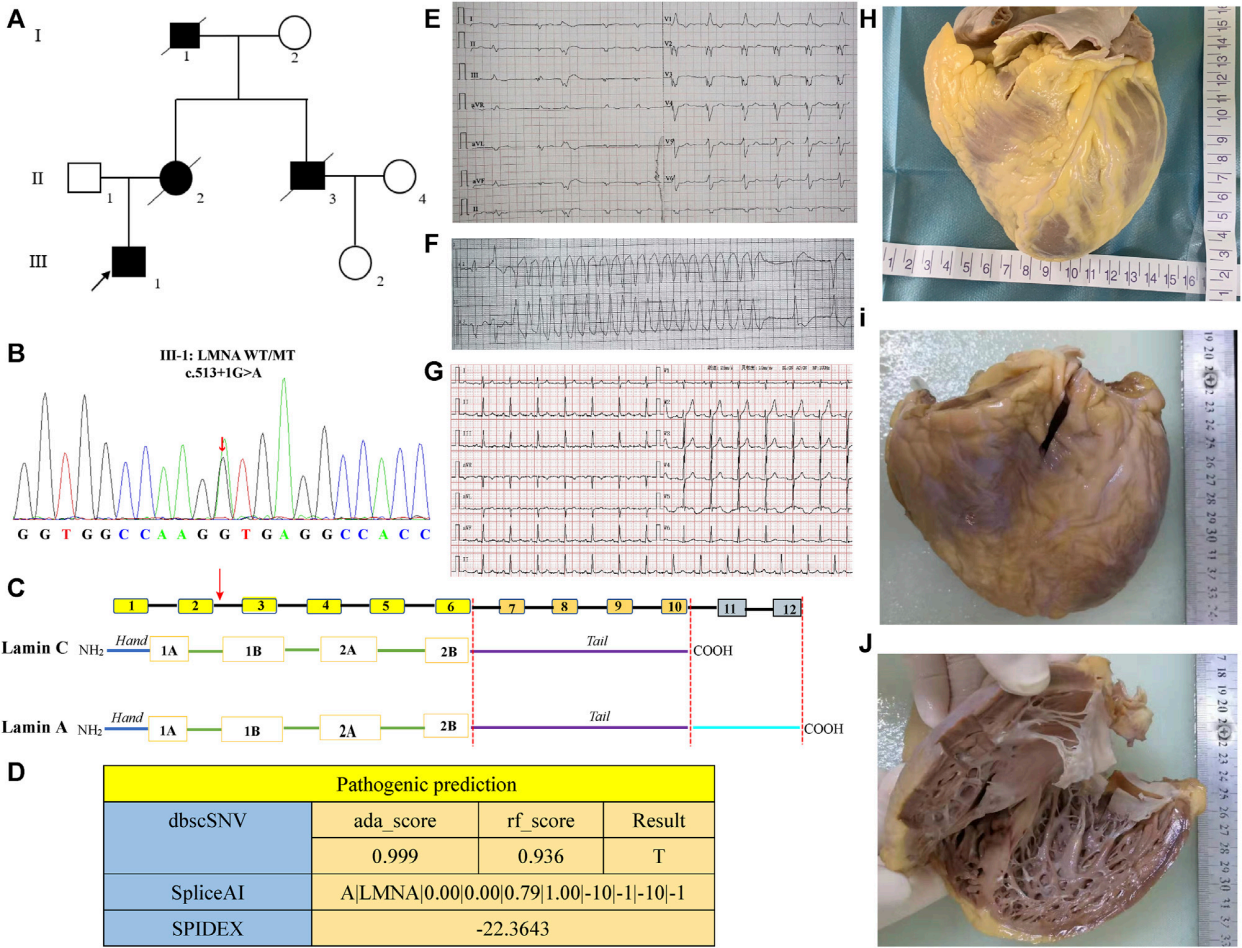
Clinical characteristics and genetic analysis of the *LMNA* mutation. **(A)** Schematic pedigree of the family. The reviewed patients (I1, II2, II3) had died of cardiac disease in their early years. **(B, C)** A heterozygous mutation of “G” to “A” transition at the splicing donor site at intron 2 (c.513 + 1 G>A). **(D)** Results of pathogenic prediction analysis by bdscSNV, SpliceAI, and SPIDEX on-line tools. **(E, F)** Electrocardiogram (ECG) of the proband before cardiac transplantation. **(G)** ECG of the proband after cardiac transplantation. **(H)** Anterior view of the abandoned healthy donor heart. **(I)** Anterior view of the proband’s heart. **(J)** Left ventricular internal view of the proband’s heart.

A 31-year-old male proband presented with symptoms that included post-activity shortness of breath, palpitation, and syncope without muscular strength involvement. The first clinical evaluation was performed in 2021, at which time ECG showed atrial fibrillation, paroxysmal ventricular tachycardia, and frequent ventricular premature diffuse ventricular block (Figures 1E,F). Echocardiography revealed the following: left atrium, 56 mm; right atrium, 57 mm; left ventricle, 60 mm; right ventricle, 29 mm; interventricular septum, 7 mm; left ventricular free wall, 7 mm; left ventricular apex, uncompacted; thickened myocardium, 5-mm thick; and left ventricular ejection fraction, 29%. Additionally, the level of blood B-type natriuretic peptide was increased (3,319 pg/mL), whereas that of creatine kinase was normal (42 U/L). During hospitalization, the patient experienced recurrent ventricular tachycardia, which was not controlled by lidocaine, propafenone, and amiodarone, and necessitated electrical cardioversion. Later, intracardiac electrophysiological examination revealed that the lesion was located in the tricuspid valve ring (one o’clock His bundle), but radiofrequency ablation was unsuccessful in two hospitals. To prevent SCD, an ICD was implanted to the proband after 1 year. The proband was repeatedly admitted to the hospital due to heart failure and had poor efficacy in taking anti-heart failure drugs such as sarcubatrixartan, digoxin, and diuretics. Heart transplantation was finally performed on 12 August 2021, and the postoperative electrocardiogram was normal (Figure 1G). Compared with the first donor heart, the patient’s isolated heart showed significant enlargement of the left ventricle, thinning of the ventricular wall, and significant noncompaction of the left ventricular apex myocardium (Figures 1H–J). It has been 2 years since follow-up, and the proband’s cardiac function has been good without any episodes of ventricular arrhythmias. The familial tree reconstructed based on verbal testimony contains six people across three generations (Figure 1A). Except for the proband, the other three family members suffered from SCD, which exhibited an autosomal dominant mode of inheritance.

### Clinical findings of 15 *LMNA* splicing mutation sites

**FIGURE 2:**
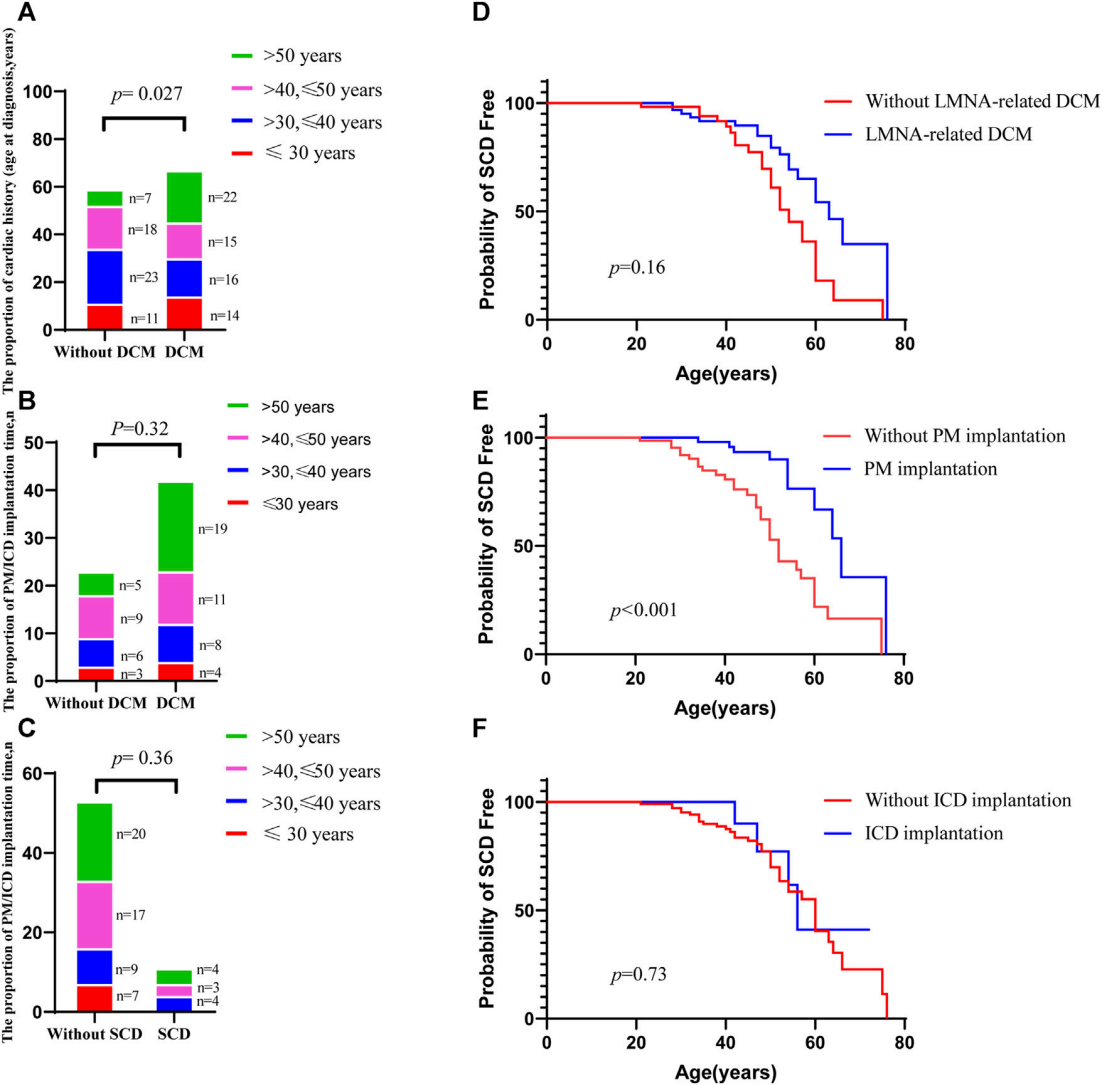
Summary of the clinical characteristics of LMNA splicing mutation sites. **(A)**. Bar chart comparison of age at diagnosis between the DCM and non-DCM groups; **(B)**. Bar chart comparison of the proportion of PM/ICD implantation between the DCM and non-DCM groups; **(C)**. Bar chart comparison of PM/ICD implantation between the SCD and non-SCD groups; **(D)**. SCD-free survival comparing the patients with DCM with those without DCM; **(E)**. SCD-free survival comparing the patients with PM implantation with those without PM implantation; **(F)**. SCD-free survival comparing the patients with ICD implantation with those without ICD implantation. *p* values of a, b, and c were calculated by Fisher’s exact test. *p* values of c, d, and f were calculated by log-rank test.

A total of 130 individuals from 17 families were enrolled and divided into the non-DCM group and the DCM group. Table 1 summarizes the main clinical features of *LMNA* splicing mutation associated with cardiac phenotype. There was no gender difference between the two groups (*p* = 0.6). The average age at onset was 42.7 ± 13 years in the DCM group and 39.3 ± 11.3 years in the non-DCM group. Compared with the non-DCM group, the DCM group presented a lower incidence of AVB (*p* = 0.015) and higher incidence rates of non-sustained ventricular tachycardia (*p* = 0.004) and ICD implantation (*p* = 0.005). A total of 10.4% of the patients underwent heart transplantation, and they were all from the DCM group. The manifestation of cardiac phenotype centered around the age of 30–40 years in the non-DCM group, and after the age of 50 years in the DCM group. There was a difference in the age distribution between these two groups (*p* = 0.027) (Figure 2A). The number of individuals who underwent PM/ICD implantation was higher in the DCM group than in the non-DCM group, but within each of the groups, there was no statistically significant difference in the age distribution (*p* = 0.32) (Figure 2B). Although the number of PM/ICD implantations was significantly higher in the non-SCD group than in the SCD group (*p* = 0.004), there was no difference in the age distribution between DCM group and without DCM group (*p* = 0.36) (Figure 2C). Furthermore, we constructed survival curves to investigate the relationship between DCM, PM implantation, ICD, and the occurrence of SCD. The results indicated that there was no difference in the occurrence of SCD depending on the presence or absence of DCM (log-rank *p* = 0.16) (Figure 2D). However, PM implantation significantly reduced the incidence of SCD (log-rank *p* < 0.001) (Figure 2D), suggesting that PM treatment for AVB prevented the occurrence of SCD. ICD primarily aims to reduce the incidence of SCD by treating ventricular tachycardia and ventricular fibrillation. Surprisingly, ICD implantation did not reduce the occurrence of SCD in patients with LMNA splicing mutation (log-rank *p* = 0.73) (Figure 2F).

**TABLE 1:** Clinical characteristics of LMNA splicing mutation associated with cardiac phenotype.

	All LMNA subjects n=(134)	Without LMNA-related DCM (n = 56)	LMNA-related DCM (n = 78)	*p*-value
Gender (male) (n, %)	78 (58.2)	34 (55.7)	44 (60.3)	0.6
Cardiac history (age at diagnosis) (years)	41.2 ± 12.3	39.3 ± 11.3	42.7 ± 13	0.12
Combined LGMD1B (n, %)	31 (23.1)	18 (29.5)	13 (42)	0.15
AVB (n, %)	84 (62.7)	45 (73.8)	39 (53.4)	0.015
Atrial fibrillation (n, %)	31 (23.1)	13 (21.3)	18 (24.7)	0.65
Non-sustained ventricular tachycardia (n, %)	28 (20.9)	6 (9.8)	22 (30.1)	0.004
PM interventions (n, %)	58 (43.3)	25 (41)	33 (45.2)	0.62
ICD interventions (n,%)	16 (11.9)	2 (3.3)	14 (19.2)	0.005
PM/ICD interventions (age at diagnosis) (years)	47.1 ± 11.8	44.5 ± 8.8	48.5 ± 13.1	0.2
SCD (n, %)	42 (31.3)	21 (34.4)	21 (28.8)	0.48
SCD (age at diagnosis) (years)	47.6 ± 12.8	48 ± 11.9	47.3 ± 13.9	0.88
Heart transplant (n, %)	14 (10.4)	0	14 (19.2)	<0.001
Heart transplant (age at diagnosis) (years)	46.6 ± 13.5	0	46 ± 13.5	<0.001

DCM, dilated cardiomyopathy; LGMD-1B, limb girdle muscular dystrophy type 1B; AVB, atrioventricular block; ICD, intracardiac defibrillator; PM, pacemaker; SCD, sudden cardiac death.

### Genetic and functional analysis

The intronic splicing mutation *LMNA* (c.513 + 1G>A) was identified (chr1:156100565, NM_170707) by WES and Sanger sequencing technology, and Sanger sequencing further verified a heterozygous mutation of “G” to “A” transition at the splicing donor site at intron 2 (c.513 + 1 G>A/wild type, Figure 1B). Furthermore, the scores of dbscSNV were ada_ Score 0.9999 and rf_ Score0.936, indicating a high probability of a harmful splicing effect. The predicted result of SpliceAI for the *LMNA* (chr1:156100565) mutation is A |*LMNA*|0.00|0.00|0.79|1.00|–10|–1|–10|–1, meaning that after G mutation A, the probability of 156100555 being a splicing receptor increases by 0.00, while the probability of 156100555 being a splicing donor increases by 79%; the probability of using 156100564 as a splicing acceptor decreases by 0.00, while the probability of using it as a splicing donor decreases by 100%. Additionally, the SPIDEX prediction result was −22.3643 (absolute value ≥2 indicates broken splicing) (Figure 1D).

### *LMNA* RNA expression analysis

**FIGURE 3:**
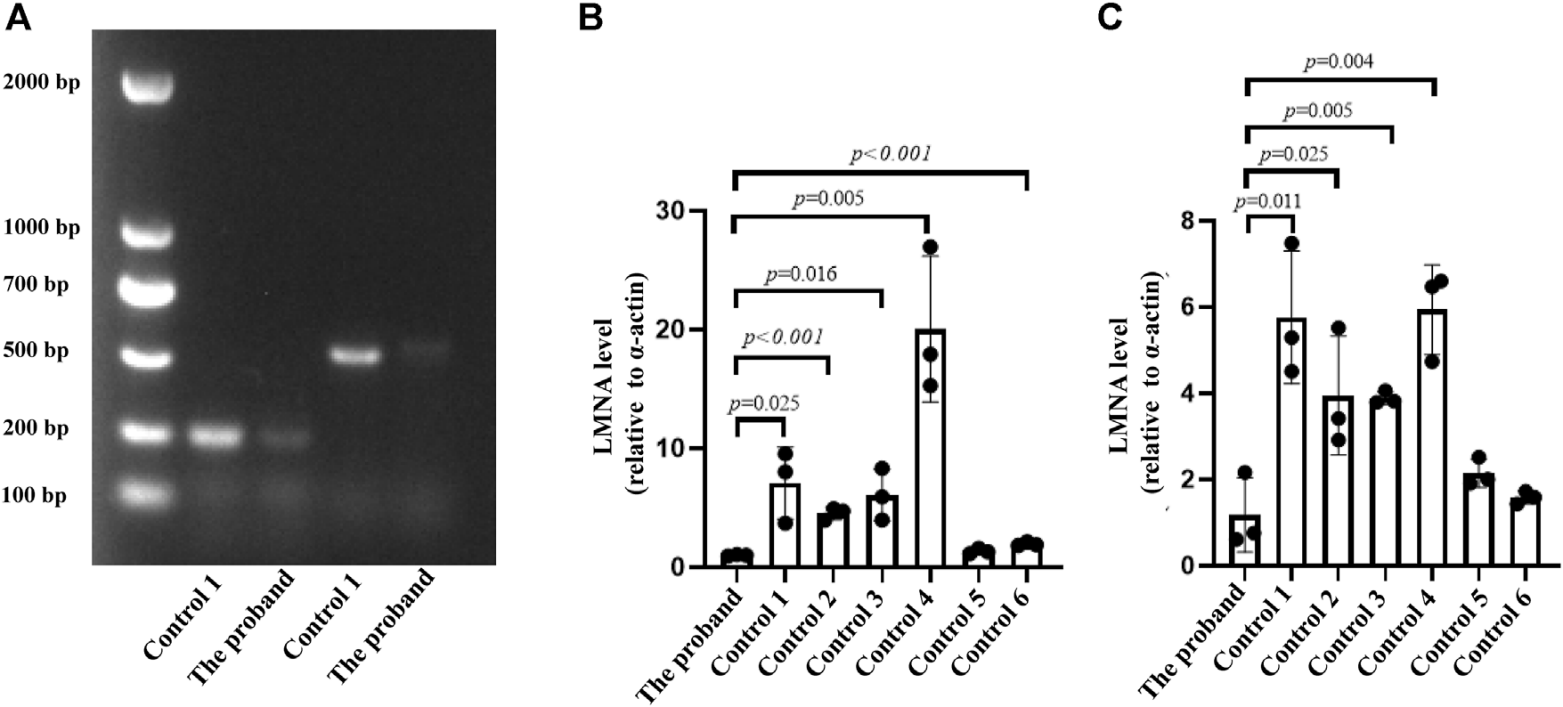
**(A)** PCR map. The leftmost strip is the DNA molecular weight marker. The 232 bp and 497 bp fragments on 1.5% agarose gel are positive for the map using the two primers. **(B, C)** Two qPCR primers were applied to detect the RNA expression of the proband and six control individuals; *n* = 3.

To understand the effect of splicing mutation on the second exon subsequence, we next sought to obtain the RNA sequence after mutation. We designed two pairs of primers in the first and third exon regions, 232 and 497 bp, respectively. After extracting RNA from peripheral blood mononuclear cells, reverse transcription cDNA was performed. However, the PCR map of the proband was very weak (Figure 3A), and no mutation sequence was detected in the sequencing of two PCR products. The sequencing results of the proband and two control groups are shown in Supporting Material (Files 1–3). Furthermore, real-time PCR evaluation of mRNA expression in the peripheral blood of the proband and six healthy individuals was performed to identify whether *LMNA* RNA expression was reduced after *LMNA* mutations. We designed the *LMNA* 11–12 Exon (11F: 5′-GCT​CTT​CTG​CCT​CCA​GTG​TC-3´; 12R: 5′-GGG​TTA​TTT​TTC​TTT​GGC​TTC​A-3′) from primer I as a specific sequence of lamin A, and *LMNA* six to seven Exon (6F: 5′-TGG​ACG​AGT​ACC​AGG​AGC​TT-3´; 7R: 5′-AGT​TTG​CGC​TTT​TTG​GTG​AC-3′) from primer II as a common sequence of lamin A/C. The results demonstrated that the patients with *LMNA* mutations showed significantly reduced LMNA transcription (*p* < 0.001, Figures 3B,C).

### Pathological analysis

**FIGURE 4:**
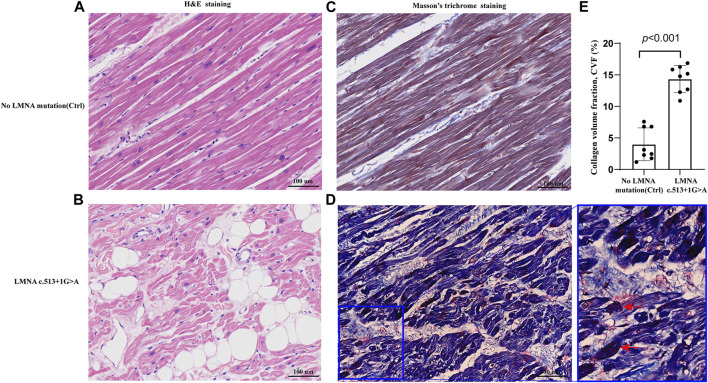
Representative sections of the control and proband hearts assessed by hematoxylin and eosin (H&E) staining **(A,B)** and Masson’s trichrome staining **(C,D)**.Homogeneous red stained coagulation particles in the cardiomyocytes of the proband (red arrows) **(D)**. Quantification of collagen volume fraction (CVF) (*p* < 0.001) between the two groups **(E)**.

**FIGURE 5:**
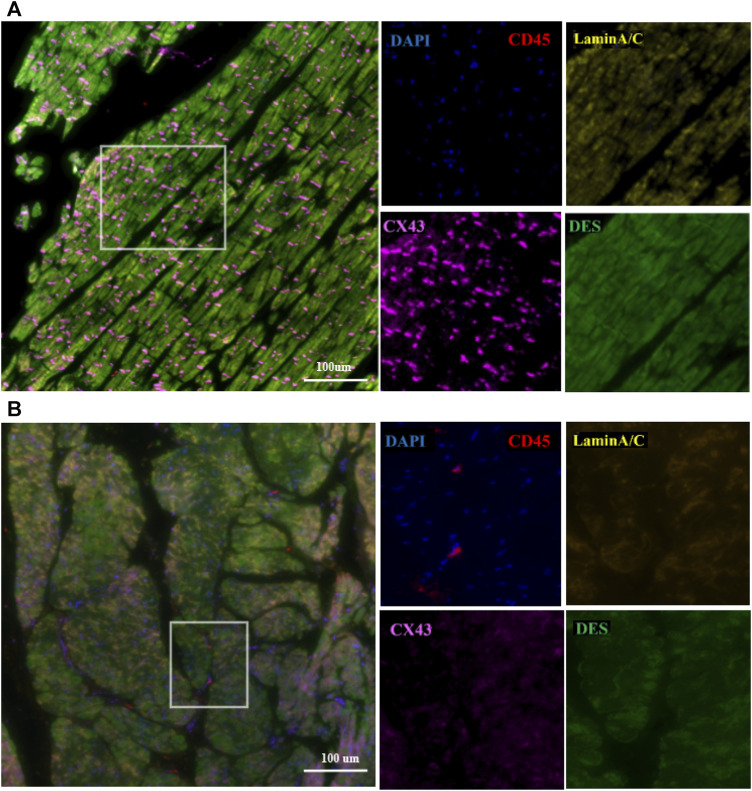
Representative polychromatic immunofluorescence myocardial staining for CD45 (red), desmin (green), Cx43 (orange), lamin A/C (yellow), and DAPI (blue). In the control group **(A)**, the distribution of DES, Cx43, and lamin A/C was uniform. In the proband group **(B)**, the distribution and expression of DES, Cx43, and lamin A/C were abnormal, and there was significant white blood cell infiltration of the myocardium. Scale: 100 μm.

**FIGURE 6:**
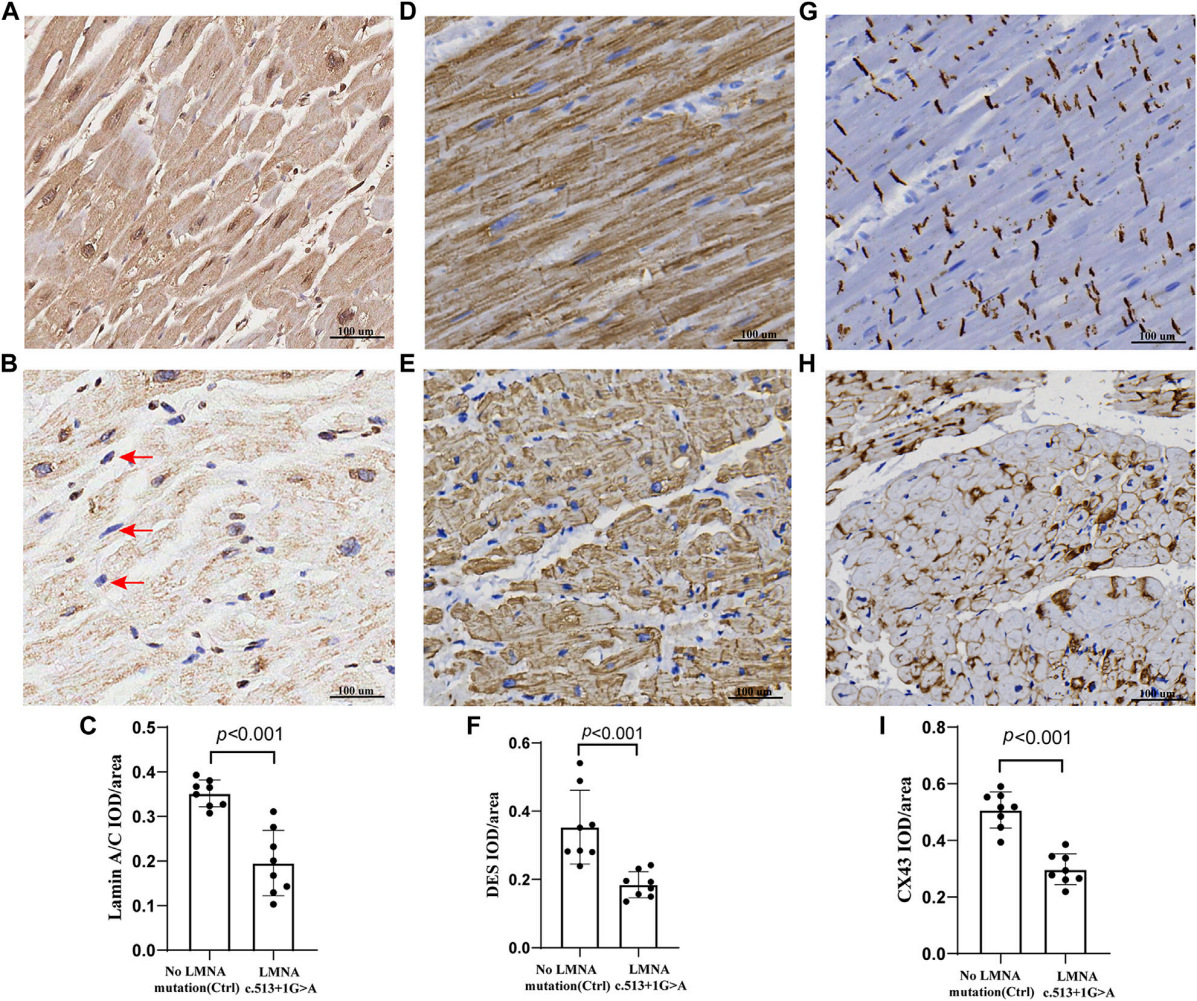
Representative sections of the control and proband hearts were assessed by lamin A/C, DES, and Cx43 **(A,B,D,E,G, H)**. Selective loss and irregularity of lamin A/C expression in myocyte nuclei (red arrows) of the proband. The relative lamin A/C protein IOD/areas are shown **(C,F, I)**. There was a significant difference in the lamin A/C, DES, and Cx43 protein integrated optical density (IOD)/area (*p* < 0.001) between the two groups. Scale: 100 μm. The results are expressed as the mean ± SEM of slices of eight regions of the left ventricular per group.

The control and proband’s left ventricular apex were assessed by various histopathologic patterns. Compared with the control group, the DCM group showed significantly more uneven hypertrophy, a larger nucleus, irregular karyotype, massive atrophy, and vascular degeneration of myocardial cells according to the results of H&E staining (Figures 4A,B). Moreover, we noted that the proband’s myocardial cells were stained blue due to the increase in collagen fibers and obvious scarring of fibers, while the normal myocardium was stained red by Masson–Goldner trichrome. Additionally, some hypertrophic cardiomyocytes showed homogeneous red-stained coagulation particles of different sizes, suggesting the remnants of incompletely decomposed protein components in myocardial cells (Figures 4C,D). DES is located in the cytoplasm, Cx43 is located in the intercalated disk, lamin A/C is located on the nuclear membrane, and CD45 (a white blood cell marker) is located in the myocardial interstitial tissue. According to polychromatic immunofluorescence staining analysis (Figure 5), the distribution and expression of DES, Cx43, and lamin A/C showed significant abnormalities in the proband group compared with the control group. Additionally, there was more significant white blood cell infiltration in the myocardium of the proband, indicating a close relationship between LMNA-related DCM and inflammatory injury. To better understand the effect of *LMNA* mutation on cytoskeletal protein and intercalated disc function, we conducted a study on the distribution and expression levels of lamin A/C, desmin, and Cx43. *LMNA* mutation leads to selective loss and irregularity of lamin A/C expression in myocyte nuclei and exhibits an aggregated distribution of desmin and partial absence of expression or aggregation to one side of Cx43. As expected, the expression levels of lamin A/C, desmin, and Cx43 were significantly lower than those of the control group (Figure 6).

## Discussion

Together, lamin A/C protein and lamin B protein, encoded by the *LMNB* gene, form the nuclear lamina, which plays a key role in the cell cycle, differentiation, signal transduction, apoptosis, DNA repair, and other cell life processes (Taghizadeh et al., 2019). In general, the two nucleotides directly connected to the exon in the splicing region are highly conservative. The bases of the 5′end (donor site) and 3′end (recipient site) of the intron are almost GT and AG, called GT-AG rules. In mammalian genomes, the probability of splicing sites with classic GT-AG combinations is 98.71% (Burset et al., 2001), and splicing mutations located in this conserved region are highly probably pathogenic. The canonical intrinsic splicing mutation in the *LMNA* gene would cause cardiac involvement with variable myopathy (Rogozhina et al., 2013; Chen et al., 2013; Burset et al., 2001). However, we report for the first time that the intrinsic variant c.513 + 1G>A exhibits a cardiac phenotype without skeletal muscle involvement. The difference in clinical phenotype is closely related to the individual difference in the fidelity of the splicing mechanism, which may determine the proportion of normal and mutant lamin A/C isoforms in cells, thus related to the severity of clinical symptoms. Mutations in *LMNA* intron and splice-related conservative sites can lead to abnormal expression and function of lamin A/C products, and we further confirmed that both the proband’s peripheral blood *LMNA* mRNA expression and the expression levels of lamin A/C on the nuclear membrane of heart slices were significantly lower than those of the control group. To investigate the impact of RNA splicing mutations on the mRNA exon 2 sequence, we extracted peripheral blood RNA from the proband and control groups for RT-PCR followed by cDNA sequencing. However, the sequencing results only showed unmodified *LMNA* sequences. Similar to the *LMNA* c.357–2A>G study (Zaragoza et al., 2016), although the *LMNA* c.513 + 1G>A mutation is predicted to result in transcription of aberrant mRNAs with an out of frame deletion of exon 2 and introduction of a premature termination codon, we found no evidence for aberrant *LMNA* splicing. We speculate that the reason for this is that the abnormal mRNA transcription of *LMNA* heterozygous mutation alleles is eliminated through nonsense mediated mRNA decay (Narula et al., 2012; Al-Saaidi et al., 2013). Although we did not obtain direct evidence of the *LMNA* mRNA sequence after splicing mutation, we used Mutation and Bioinformatics Analysis tools to predict pathogenicity. However, the clinical diagnosis of rare genetic disorders still faces many problems: the diagnosis rate of exon group sequencing is only 25%–30%; the sequence of the non-coding region obtained by genome sequencing can account for 90%, but the mutation of the non-coding region that destroys the normal splicing mode of mRNA is often ignored because it is difficult to accurately identify. The artificial intelligence software Splice AI can accurately predict the splicing change caused by single nucleotide variation. In patients with neurodevelopmental disorders, approximately 10% of pathogenic mutations may be spliced non-coding mutations, which will lead to an abnormal splice and abnormal splicing point mutations often leads to a variable splice (Jaganathan et al., 2019). In brief, the predicted pathogenicity of *LMNA* c.513 + 1G>A with splicing mutations is consistently extremely harmful, as determined by SpliceAI, dbscSNV, and SPIDEX tools.

Our study revealed that *LMNA* splicing mutations resulted in *LMNA*-related DCM in 58.2% of cases and AVB in 62.7% of cases, which is markedly higher than the incidence rates of 6.2% and 32% reported for *LMNA* missense mutations in another study (Hasselberg et al., 2018). Apart from AVB, non-sustained ventricular tachycardia (occurring in 23.1% of cases) and advanced heart failure were the main causes of SCD. Guidelines recommend considering an ICD in the presence of two of the following risk factors: left ventricular ejection fraction <45%, male sex, non-sustained ventricular tachycardia, and nonmissense variants (Al-Khatib et al., 2018). Wahbi et al. constructed a new risk prediction score for life-threatening ventricular tachyarrhythmias (LTVTA) in laminopathies, and proposed that a threshold between 7% and 10% at 5 years represented a satisfactory compromise between the identification of the maximum number of patients with LTVTA and the minimization of unnecessary ICD implantations (Wahbi et al., 2019). However, ICD implantation did not decrease the occurrence of SCD in the patients with LMNA splice mutations. Undeniably, the statistical bias observed in this study might be attributed to the limited sample size. Another critical factor is that with advancing age, there was a significant increase in the number of individuals with LMNA-related DCM. SCD resulting from refractory heart failure renders ICD implantation ineffective as a preventive measure. Actually, nearly half of presumed SCDs are not arrhythmic (TSeng, et al., 2018), and less than 30% of SCD cases are due to shockable ventricular arrhythmias, while trends indicate that asystole and pulseless electrical activity are the more common presenting rhythm among SCD victims in the general population (Khairy et al., 2022). Therefore, relying solely on ICD for preventing SCD was confirmed insufficiently effective. Cardiac resynchronization therapy implantable defibrillator (CRT-D) is the preferred treatment for severe heart failure, cardiac dyssynchrony, and ventricular arrhythmia survivors. It has been demonstrated that CRT-D could improve systolic function, associated with survival benefits in in patients with LMNA-related DCM during a median follow-up of 1.3 years (Sidhu et al., 2022). However, further clinical studies are needed to confirm whether CRT-D effectively reduces the long-term cardiac mortality rate in patients with LMNA-related DCM. Our research confirmed that PM implantation can reduce the incidence of SCD, with the primary beneficiaries being LMNA-related non-DCM patients with concurrent AVB. There is currently no consensus on the indications for heart transplantation in patients with LMNA-related DCM. We found a heart transplantation rate of 10.4% among the patients with LMNA-related DCM, with no reported instances of SCD, indicating the therapeutic value of heart transplantation. However, Hasselberg et al. reported that 19% of LMNA patients required heart transplantation, and the combined incidence of mortality or heart transplantation over nearly 8 years of follow-up was 24% (Hasselberg et al., 2018). The limited availability of heart donors, high costs, and postoperative complications such as infections and bleeding have hindered the widespread adoption of heart transplantation.

The myocardial cytoskeleton and related proteins form the structural basis of myocardial cell contraction. Once the cytoskeleton components of myocardial cells are damaged, the stability of the sarcomere of the myocardium will be damaged. Unstable sarcomere movement will lead to myocardial contraction disorder and affect intracellular signal transmission. Therefore, skeletal protein defects represent an important mechanism of DCM (Le Dour et al., 2022). However, the molecular pathogenesis of *LMNA*-related DCM has not yet been clearly clarified. It has been widely proposed that *LMNA* deletion or mutation can cause weakness of the nuclear lamina skeleton protein and structure, damage to the cardiomyocyte coding sarcomere or Ca^2+^ processing protein, and lead to cardiomyocyte apoptosis and abnormal contractile function, which has been implicated in disease progression in DCM and arrhythmias (Nikolova et al., 2004; Su et al., 2022; Chatzifrangkeskou et al., 2023). Lamin A/C is localized in the nuclear and nucleoplasm, and desmin can transiently enter the nucleus and interact with transcription factors by connecting lamin A/C (Fuchs et al., 2016). Moreover, lamin A/C deficiency causes desmin detachment at the nuclear–cytoskeletal interface. Changes in the spatial structure of desmin can destroy the fiber skeleton, which will lead to an increase in the mechanical fragility of myocardial fibers and eventually lead to the collapse of the myocardial cell structure. Additionally, lamin A/C knockout mice show disorganization of desmin nuclear attachments, a Z disc cross-striation pattern, and variation in the intensity of myofibril staining (Nikolova et al., 2004). We found similar results in the cardiac pathological biopsy of the proband, showing that *LMNA* splicing mutations could cause myocardial colonization and interstitial fibrosis and decrease the expression level of desmin with uneven accumulation distribution. Gap junction channels, mostly formed by CX43, are important for desmin anchoring intercalated discs, which allow electrical coupling between neighboring cardiomyocytes. It was found that remodeling of gap junctions represented redistribution to the lateral sides of cardiomyocyte membranes (i.e., “lateralization”) and reduced expression of Cx43 in the heart of LMNA^H222P/H222P^ (H222P) mice (Macquart et al., 2019). Additionally, our study showed that 23.1% of the patients with LMNA splice mutations concurrently presented with atrial fibrillation, and the latest research has confirmed that atrial fibrillation is related to decreased expression and lateral distribution of Cx43, as well as interstitial fibrosis (Shin et al., 2015). We further confirmed that the *LMNA* splicing mutation caused myocardial electrical activity disorder and was closely associated with intercalated disc remodeling and downregulation of Cx43 expression. LMNA mutation also displayed aberrant calcium homeostasis, which led to arrhythmias at the single-cell level, representing another potential pathogenesis of *LMNA*-related DCM (Lee et al., 2019). Moreover, we confirmed for the first time that there was a significant white blood cell infiltration of the diseased myocardium with *LMNA* mutation. Previous studies have shown that disruption of nuclear membrane integrity can lead to immune inflammatory response and activation of autophagy (Yao et al., 2021). However, further research is needed to clarify how inflammatory damage participates in the pathogenesis of *LMNA*-related DCM.

## Conclusion

First, we summarized for the first time the clinical characteristics of the LMNA splicing mutation–related cardiac phenotype. In our cohort, implanting a pacemaker significantly reduced the SCD rate in non-DCM patients with AVB, while the sole implantation of an ICD was not effective in reducing the SCD rate in DCM patients with non-persistent ventricular tachycardia and heart failure. Second, we used LMNA c.513 + 1G>A as an example to study the pathogenesis. We demonstrated that *LMNA* c.513 + 1G>A not only downregulated the expression of normal *LMNA* mRNA but also affected the expression of cytoskeletal proteins in myocardial cells. We comprehensively summarized the pathological characteristics of *LMNA*-related DCM, including hypertrophy, atrophy, fibrosis, inflammatory cell infiltration, intercalated disc remodeling, and downregulation of desmin and Cx43 expression. We propose that the disordered myocardial cytoskeleton protein and the impairment of intercalated disc function may be the underlying molecular mechanism of DCM and ventricular arrhythmia caused by *LMNA* c.513 + 1G>A. Statistical limitations stem from our utilization of a retrospective clinical cohort study, coupled with the possibility of statistical bias due to the relatively small sample size. Further large-sample, multicenter research data are necessary to validate the outcomes we have derived. Future works should focus on the potential regulatory mechanism of *LMNA* splicing mutations on skeletal proteins. We hope that the results will help us to understand the pathogenic characteristics of *LMNA*-related DCM.

## Data Availability

The original contributions presented in the study are publicly available. This data can be found here: https://databases.lovd.nl/shared/phenotypes/0000326855.
